# Concept-formation and deep disagreements in theoretical and practical reasoning

**DOI:** 10.1007/s11229-024-04884-6

**Published:** 2025-01-20

**Authors:** Michael Wee

**Affiliations:** Department of Psychiatry, University of Oxford, Warneford Hospital, Oxford, OX3 7JX, United Kingdom

**Keywords:** Wittgenstein, On Certainty, Concept-formation, Deep disagreements, Practical reasoning, Moral disagreement

## Abstract

This paper explores the idea that deep disagreements essentially involve disputes about what counts as good reasoning, whether it is theoretical or practical reasoning. My central claim is that deep disagreements involve radically different paradigms of some principle or notion that is constitutively basic to reasoning—I refer to these as “basic concepts”. To defend this claim, I show how we can understand deep disagreements by accepting the indeterminacy of concept-formation: concepts are not set in stone but are responsive to human needs, and differences in individuating and ordering concepts lead to clashes in paradigms of reasoning. These clashes can be difficult to resolve because linguistic concepts, especially basic concepts, impose a normative structure onto thought to make reasoning possible at all. This, I also argue, is an authentically Wittgensteinian account of the nature of reasoning. While deep disagreements involving theoretical and practical reasoning both stem from the same root problem of clashing paradigms of basic concepts, I will also draw attention to the particularly radical indeterminacy of moral concept-formation, which makes moral deep disagreements more difficult to resolve. Over the course of the paper, I will discuss two examples of deep disagreements to illustrate and defend my central claim: deep disagreements over vaccines and the concept of “evidence” (theoretical reasoning) and deep disagreements over affirmative action and the concept of “fairness” (practical reasoning). I conclude by suggesting how my account of reasoning does not lead to moral relativism.

## 1 Introduction

The classic account of deep disagreements goes something like this: there are some disagreements that are not amenable to rational resolution. This irresolvability must not be due to some defect on the part of the disputants—as Fogelin (1985, p. 5) writes in his seminal article, “The Logic of Deep Disagreements”, an argument might be irresolvable simply because “one of us is dense or pig-headed”. Rather, the irresolvability is due to “a clash in underlying principles”. Such disagreements we can call deep disagreements. They are to be contrasted with ordinary disagreements, where disputants still have sufficient common ground between them by way of shared beliefs or principles, such that they can marshal reasons in the hope of resolving the dispute. In deep disagreements, “the conditions for argument do not exist” because that sort of shared background does not exist.

Notably, Fogelin (ibid., p. 6) also connects this account to an exegetical claim, namely that these ideas about deep disagreements come from Wittgenstein’s *On Certainty*. He quotes *On Certainty* to make the point that in deep disagreements we are “using our language-game as a base from which to *combat*” an entirely different language-game (Wittgenstein, 1977, § 609), and thus reasons do not go very far. “At the end of reasons comes *persuasion*” (ibid., § 612). This seems to imply that deep disagreements concern the very nature of reasoning and its outer limits.

There are details of Fogelin’s account which need finetuning and modification, but it is this implication about deep disagreements that I think is most important and worth preserving. In this paper, I want to explore how we can characterise deep disagreements as essentially involving disputes about what counts as good reasoning, whether this is theoretical reasoning or practical reasoning. Typically, deep disagreements relating to the latter will be what we might call “moral deep disagreements”, such as those over affirmative action or abortion, whereas deep disagreements concerning theoretical reasoning are often over scientific matters, like vaccine-scepticism or the flat earth movement. Of course, a single issue may involve disputes about concepts from both types of reasoning.

My working hypothesis will be that deep disagreements involve a radical difference in the understanding and use of some principle or notion that is constitutively basic to reasoning—for ease of reference I will call such putative principles or notions “basic concepts”, and the central case(s) of understanding and using such concepts their “paradigm”. I will consider as candidates for basic concepts terms like “evidence” (theoretical reasoning) and “fairness” (practical reasoning). But the paradoxical mark of a deep disagreement is this: while two disputants may possess such different versions of a basic concept that one cannot be corrected by the standards of the other, yet they can typically still understand each other—and be understood by others—as talking about “the same” linguistic concept, at least in a loosely analogous sense.

Deep disagreements will, on my account, be immune to *ordinary* means of rational resolution like giving evidence or providing justification, because for a concept to be truly constitutively basic to reasoning it cannot be proved by evidence or justified according to criteria *external* to it. But if the question of justifying basic concepts is not open to rational inquiry, then in order to better understand the nature of deep disagreements and how it may even be possible to resolve them, we need to ask a different set of questions: how exactly do basic concepts shape and constrain our reasoning? Can we change them at will? These are questions about the nature of reasoning and of linguistic concepts which I shall attempt to answer in this paper. In doing so I shall also explore the question of whether there are *extraordinary* means of rational resolution for deep disagreements—where someone adopts a new paradigm of reasoning not simply under duress or some other non-rational force, but by a process we can still justifiably consider rational, in a more capacious sense of the word.

Let me make two more preliminary points about my approach. Firstly, the account of reasoning and linguistic concepts that I will develop and defend here is, I believe, an authentically Wittgensteinian one. This paper is not primarily a work in exegesis, and that is in part because Wittgenstein gives fertile suggestions on this topic but not fully worked out answers. So my account will involve a development, and not a strict reconstruction, of Wittgenstein’s thought. Secondly, it is an important feature of my account that deep disagreements about scientific and moral matters stem from the same root problem of radically different basic concepts of reasoning. However, over the course of my argument I will also point out some distinct issues raised by deep disagreements involving practical reasoning, which give moral deep disagreements a particularly intractable feel.

My argument will proceed as follows. In Sect. 2, I will discuss the distinctiveness of my hypothesis in relation to the recent literature on deep disagreements. In Sect. 3, I will illustrate its plausibility with two examples—one scientific, one moral—of deep disagreements, and give a preliminary assessment of what I take to be the four key features of basic concepts. On the basis of this discussion, in Sect. 4, I will develop a more detailed account of reasoning in relation to linguistic concepts, drawing on Wittgenstein’s thought. In Sect. 5, I will apply this account back to deep disagreements to produce a more fine-grained analysis of the rational nature of such disputes and, potentially, their resolution. Finally, in Sect. 6, I will consider the problem of a relativism of reasoning, especially in practical reasoning, and suggest how this can be defused.

## 2 A problem of rational evaluation

I want to begin with a discussion of the distinctiveness of my hypothesis—that deep disagreements are clashes about what it means to reason well, due to different paradigms of key concepts in reasoning—vis-à-vis both the older and the more recent literature on deep disagreements, before arguing for its plausibility in the next section.

Earlier discussions of Fogelin’s article have centred around the nature of rationality and argumentation, with a particular focus being the rational resolvability of deep disagreements and whether Fogelin was right to be pessimistic about this (see, for example, Lugg, 1986; Davson-Galle, 1992; Feldman, 2005). However, much of this discussion is not particularly Wittgensteinian and the role of concepts in shaping the practice of reasoning, as I will be proposing in this paper, is not discussed to my best knowledge. Nonetheless, these earlier discussions were right to identify the question of resolvability as a key issue—if a disagreement is rationally irresolvable in principle because of a lack of shared background as Fogelin suggests, then it would be difficult to make sense of it as a bona fide *disagreement* in the first place, rather than an incommensurable difference in systems of thought. But if these disagreements are resolvable by rational means, though perhaps more slowly than ordinary disagreements, then they are truly “no cause for alarm” as Lugg (1986) suggests, in an early critique of Fogelin.

I believe there is a way out of this dilemma about characterising deep disagreements. Part of this paper’s aim will be to show how a fine-grained understanding of the way certain concepts constitute and shape the nature of reasoning can both do justice to the idea that some disagreements are indeed deep and irresolvable in ordinary ways, and yet make sense of them as genuinely rational disagreements. In this way, my position straddles the optimist-pessimist divide proposed by Aikin (2019) in his taxonomy of responses to deep disagreements—like the pessimists I will argue for the genuineness of deep disagreements, and like the optimists I will argue for their rational resolvability.

Turning to more recent discussions of deep disagreements, it is striking that the nature of reasoning and its role in explaining deep disagreements have been given less attention. The root of this neglect, I would argue, is the shift towards treating deep disagreements as a problem of epistemology (see, for example, Lynch, 2016; Pritchard, 2018 and 2021; Ranalli, 2020) rather than of informal logic, as in the case of Fogelin and his early commentators. I am not suggesting, of course, that we should draw a sharp distinction between epistemology and informal logic, between understanding deep disagreements as a problem of knowledge and justification versus as a problem of reasoning. The two types of philosophical problems are intimately related. Indeed, various epistemological accounts have offered explanations of how deep disagreements involve different standards or grounds of reasoning, leading to radically different knowledge claims. But as I will elaborate subsequently, it is not simply standards or grounds of reasoning that may differ; a key part of my Wittgensteinian account of reasoning is that the very notion and practice of reasoning is plural and not stable. Reasoning depends on the concepts that constitute it, and these concepts provide more than just standards of evaluation but substantial normative structures of thought (see Sect. 4).

To illustrate how my perspective differs from those of recent discussions in the literature, let us look at a few key examples. Ranalli and Lagewaard (2022) helpfully classify three different views in the recent literature about the kind of shared background that makes rational argumentation possible—and the absence of which helps to explain the phenomenon of deep disagreements:

(1) Shared worldview: “overlapping set of interconnected metaphysical and ethical beliefs”;
(2) Shared epistemic principles: “shared principles which specify when a piece of evidence E supports (a disputed proposition) *p*, or how to weigh different pieces of evidence, E1, E2, E3, …, vis-à-vis the question about whether *p*”;
(3) Shared hinge commitments: “basic presuppositions of a person’s worldview or belief system, as well as the basic presuppositions that guide inquiry”.

In all of these accounts, when these beliefs, principles or commitments are not shared, people inevitably reason differently and hence come to different epistemic conclusions. Nevertheless, the trouble with the framing of “epistemology” is that it tends to assume that different parties are working within commonly accepted ideas of what reasoning looks like: the beliefs, principles or commitments principles that ground rational evaluation may differ between people, but with that ground in place, how we reason surely cannot differ wildly. This is particularly so for (1) and (2). Granted, in the case of Lynch’s (2010) account of epistemic principles and deep disagreements, which would fall under (2), it would likely include something like a difference concerning the scope and effectiveness of abductive inference—someone might dispute that this principle extends to evidence about the age of the earth and prefer to rely on literal biblical claims for this matter. This would seem to be a deep disagreement turning on a difference in what reasoning looks like. However, this still assumes a fairly stable notion of abductive inference, even if there are fundamental disagreements about its applicability. It is not as radical a claim about reasoning as the Wittgensteinian one I will put forward.

Regarding (3), the assumption that rational evaluation remains largely the same despite different starting points is, surprisingly, present even in Pritchard’s hinge epistemology approach to deep disagreements (2018 and 2021; cf. 2012). Superficially, it seems like Pritchard’s view does explain deep disagreements in terms of the plurality of standards for rational evaluation. For Pritchard, it is of great importance that rational evaluation is always local, and never universal. Rational evaluation is always relative to the range of *arational* “hinge commitments” we have. This is an intrinsic feature of the structure of reasoning—rational evaluation by its nature must be enabled by such commitments, and as such these commitments cannot themselves be subject to rational evaluation. They are therefore not propositions or beliefs, but convictions, of which we are optimally certain. Being the unspoken backdrop against which we conduct rational evaluation, they are typically mundane: I have hands, the earth is round, my name is so-and-so, etc. Under certain circumstances, of course, these certainties could be called into question (e.g. after surgery on my hands) and may require rational evaluation as potential beliefs, but normally they function as arational commitments *enabling* rational evaluation. Deep disagreements, for Pritchard, are those that involve divergent hinge commitments, e.g. about God in the case of disputes between religious believers and atheists.

But this is how Pritchard illustrates differences in rational evaluation owing to its local nature:

“…although someone raised in China, with a different name (and so on), will have hinge commitments with different contents to mine, once we abstract from these contextual differences we are effectively sharing the same hinge commitments… these differences are purely superficial, and provide no basis for thinking that, for example, we do not have shared standards of evidence, or lack shared epistemic principles.” (Pritchard, 2021, p. 1121)

So, on closer examination, what Pritchard’s view really amounts to is that hinge commitments provide different *content* that ground whole areas of rational evaluation; disputants do not substantially differ in their standards of reasoning or epistemic principles. For instance, a flat-earther would have a deep disagreement with those who are optimally certain that the earth is round. Since Pritchard emphasises that one’s hinge commitments cannot be in conflict with one’s wider beliefs (ibid., p. 1122), we can surmise that a likely cause of this divergence in hinge commitments is that the flat-earther is far more ignorant about a wide range of technical knowledge which has some connection to the shape of the earth, from beliefs about eclipses to how SatNav systems work. A flat-earther whose wider set of beliefs changes gradually may eventually come to revise her hinge commitment about the earth’s shape (ibid.). I have no doubt that ignorance accounts for some aspects of deep disagreements, but it seems too hasty to ignore the possibility that disputants may actually differ in the way they reason, and not just in the content provided by their hinge commitments. This may severely limit the possibility of changing hinge commitments even in the face of high-quality evidence.

This discussion so far, in my view, complements Lavorerio’s (2021b, p. 489) criticism of what she calls “the fundamental model” of deep disagreements—epistemological approaches that try to identify the source of deep disagreements with some fundamental level of epistemic systems, such as Pritchard’s hinge commitments. This model, argues Lavorerio, leads to an artificial “*separatedness*” between that fundamental epistemic level and “other epistemic categories, like evidence or beliefs”. As a result, Pritchard “seems to afford evidence with a neutral status” and does not consider how radically divergent understandings of a domain of knowledge will lead disputants to “accept different information as evidence”.

I share Lavorerio’s overall analysis that epistemological approaches of this kind are not fine-grained enough to deal with the possibility that deep disagreements arise from divergences at multiple levels of reasoning. The emphasis of my critique is different, however; it is not simply that different people might accept different information as evidence—people might be working from different conceptions of good evidence. This also seems to be borne out by McIntyre’s (2019) interactions with flat-earthers, whom he describes as relying a lot on first-person observation, sometimes with the help of fairly rudimentary tools, as “evidence”. He then comes to this striking conclusion:

“It’s not just that their ‘evidence’ was bad, but that they weren’t reasoning about evidence in a scientific way.” (ibid., p. 695)

This is helpful corroboration for my perspective that deep disagreements point us to a problem of plural paradigms—and not just standards—of rational evaluation. I will now turn to illustrating and arguing for the plausibility of my hypothesis, keeping in mind the need for my analysis to be sufficiently fine-grained in light of Lavorerio’s insightful critique. Basic concepts of reasoning, as I will show, do not constitute a separable fundamental level of rational evaluation. They are bound up with our experiences of reasoning.

## 3 Conceptual features of deep disagreements

Why, then, should we take seriously the possibility that deep disagreements involve radically different paradigms of reasoning well, through divergence in constitutively basic concepts of reasoning? In this section I will illustrate the plausibility of my hypothesis with two examples of deep disagreements, one scientific, one moral: vaccine-scepticism (Dare, 2014) and affirmative action (Fogelin, 1985). The former involves theoretical reasoning, the latter practical reasoning. These examples will, on further analysis, also point us to some interesting features of basic concepts of reasoning and how they account for the particular character of deep disagreements. This initial discussion will provide the basis for a more fully fledged account of reasoning and concept-formation in Sect. 4.

### 3.1 Two examples deep disagreements

When we examine deep disagreements about vaccines or affirmative action, it seems plausible to suggest that these disputes involve radically different ideas about the meaning and importance of “evidence” or “fairness” respectively, rather than a wholesale rejection of either concept from one disputant. Other important concepts will likely be involved, but for the sake of clarifying the point I will stick to one basic concept per example, without sacrificing accuracy of description:

**Vaccines:** In the case of vaccines, a scientist who appeals to statistical evidence to defend the safety record of approved vaccines may be met with wide-reaching scepticism about the reliability of evidence in relevant areas of scientific research. It is well-known that vaccine-scepticism relating to autism often involves incidents of children who reportedly first show signs of autism shortly after their first jab (cf. Davidson, 2017, p. 404; Pivetti et al., 2020, p. 7). We could imagine an inexperienced researcher being unsettled by such incidents, but being committed to scientific methods she allows herself to be corrected by more experienced scientists. The difference with the sceptic is that she has taken such incidents to become the paradigmatic instance of evidence in this and similar cases, such that she is now operating outside of the scientific conception of evidence.

So, the sceptic may take the view that the apparent directness of the connection between vaccination and the appearance of symptoms is so convincing, there must be something that the population-level statistical evidence is failing to capture. After all, she may reason, it is not impossible that in individual cases the vaccine does cause autism, and yet there is no statistical difference of the incidence rate of autism between vaccinated and unvaccinated children. There may be unknown biological factors complicating the picture, or underreporting of symptoms, or worse still a cover-up amongst scientists. As long as these remain faintly plausible options to fill out the picture, the sceptic can still say of those incidents in question, “If this is not evidence, then what is?”

**Affirmative action:** A standard way of charactering disputes about affirmative action is appealing to the distinction between “equality of opportunity” and “equality of outcome” or “result” (cf. Rosenfeld, 1985, pp. 855−857). Both may be appealed to in conversations about fairness, and it is likely that most people have room for both types of equality as part of their thinking about fairness and justice, though few will have a fully worked out conception of how to manage potential conflicts and trade-offs between the two (cf. Saito, 2013). A dispute about affirmative action may be a deep disagreement when the disputants are deeply entrenched in these different paradigms of fairness.

Imagine a policymaker whose general framework for thinking about fairness is equality of opportunity, on the basis of equal rights for individuals. She may make some concessions to equality of outcome, particularly in lower-stakes contexts or where merit is not a decisive factor. She may even try to work out how equality of outcome can be subsumed under the more important paradigm of equality of opportunity (cf. Roemer, 2003) or attempt to accommodate some modest forms of affirmative action. Taylor (2009), for example, has discussed the possibility of a Rawlsian framework of justice as fairness accommodating modest forms of affirmative action, while arguing that it will almost always reject more robust forms like quotas. So our policymaker will likely strenuously oppose robust forms of affirmative action relating to corporate boardrooms or university places.

She may, in the course of her work, come up against a politician who wants to reshape legislation to promote equality of outcome, on the basis of local and regional histories of systematic oppression, which to her justify a radical shakeup of the way resources and opportunities are currently distributed (cf. Premdas, 2016, who contrasts two paradigms of “social justice” in a broadly similar way). Suppose the politician was the sort who said, “I believe in equality of opportunity as an ideal endpoint, but we are not there yet,” we may hesitate to call this a deep disagreement. This may be a difference in strategy or a difference in interpretation of the stakes involved in university and boardroom quotas. After all, the policymaker may not have a clear position on where to draw the line between acceptable and unacceptable affirmative action (unless she is resolutely Rawlsian). But if the politician said “equality of opportunity is always a tool used by the powerful to entrench inequalities on the basis of procedural fairness” then we might have a deep disagreement pertaining to radically different paradigms of fairness.

These, I suggest, are reasonably accurate representations of views held in these two areas of deep disagreement. My account of the vaccine-sceptic’s conception of “evidence” certainly chimes with what McIntyre, as quoted in the Sect. 2, has reported of flat-earthers. But though these vignettes may provide a prima facie illustration of my hypothesis, they require more detailed elaboration regarding the nature and role of the basic concepts involved.

### 3.2 Basic concepts: a preliminary assessment

Let me thus make a preliminary assessment of the features of basic concepts and why they are so important to deep disagreements, in the light of these examples. There are, in my view, four key features—though they are really two pairs of features, as will become clear.

#### 1 Indispensability

The most obvious feature of basic concepts is that they are indispensable to reasoning. They are not laws of logic as such, but some notion of them is required for large swathes of reasoning we would not generally consider optional in human life. To engage in any kind of inductive inference is to rely on some conception, if unspoken, of evidence. Some kind of principle or set of principles relating to fairness, broadly understood, is arguably intrinsic to our reasoning about what we owe to others. By contrast, a principle like transparency—though arguably fundamental to good governance—seems less basic to practical reasoning than fairness. Arguably, it presupposes institutions or procedures which may not be culturally universal, or is a more context-specific application of more basic concepts like fairness. A disagreement about transparency, where there was a suitably shared background about principles of fairness and related concepts, would not likely strike us as being deep. This may be a dispute about the most effective or the most proportionate mechanism for transparency in government, within an accepted framework. By contrast, if the disagreement about transparency was a specific manifestation of a more fundamental dispute concerning what counts as fairness in public life, e.g. that between pro-democracy activists and a totalitarian regime, then this would likely be a deep disagreement.

#### 2 Unjustifiability

The feature of basic concepts that most directly accounts for the “depth” of deep disagreements, however, is their unjustifiability. This feature is a consequence of their indispensability. For a concept to be truly constitutively basic to reasoning it cannot be proved by evidence or justified according to criteria *external* to it. To search for such external evidence or criteria would be to attempt to step outside of reasoning, and that would no longer be a rational inquiry. This is a deeply Wittgensteinian point, that stepping outside of logic and language is impossible. In the *Tractatus*, Wittgenstein writes that “the laws of logic cannot in their turn be subject to laws of logic” (1961, 6.123). This is sometimes associated with Sheffer’s term the “logocentric predicament”: in order to articulate the foundations of logic “*we must presuppose and employ logic*” (Sheffer, 1926, p. 228).

While deep disagreements do not involve the laws of logic as such, given that they involve principles or notions that are indispensable, a similar point about unjustifiability applies. In both examples of deep disagreements, because the clashing concepts involved are sufficiently different that they cannot be corrected by the other’s standards (unlike the inexperienced researcher who makes a mistake), there is no further standpoint outside of the disagreement from which the disputants can stand and assess who has the “right” concept of evidence or fairness. To do so would already be to presuppose a concept of evidence or fairness that could adjudicate the dispute, when that very concept is in question. At most, they can each explain why their concept of evidence or fairness is a “good” one, relative to their wider system of concepts, beliefs, and practices. This is also why disputants in deep disagreements, especially in moral matters, often end up sounding like they are just confirming their biases. In fact, this is not necessarily owing to a limitation of their powers of reasoning.

#### 3 Breadth

Basic concepts are also suitably broad; they are typically formed by, and group together, more than one paradigm. Transparency is a much narrower concept than fairness, which also involves the concepts of equality, rights, and dignity, among others. Informed consent is a narrow concept with specific paradigms in healthcare and research; the principle of autonomy, which is thought to ground informed consent, is a much broader concept of practical reasoning which also extends to marriage, childcare and financial affairs, among others. The breadth of basic concepts can be seen from the possibility of great overlap in spite of a deep disagreement. In the case of vaccines outlined above, it is possible that the sceptic may still accept standard scientific evidence relating to other forms of technology and research. A moderately Rawlsian policymaker, as we have seen, may still accept some forms of affirmative action, but will have an irresolvable conflict over others when faced with someone who has a rival concept of fairness and equality.

#### 4 Indeterminacy

The breadth of basic concepts leads to a certain indeterminacy about their content. On a more mundane level, we can see that the exact term we use to discuss the relevant basic concept is probably not of minimal importance. I could easily recast the deep disagreement over vaccines as a problem of “causation” and make a case for that as an indispensable, unjustifiable, and broad concept. “Fairness” and “justice” are sometimes used interchangeably. To invoke one notion or principle is to involve a whole host of other related and overlapping concepts. But this is not a mere semantic quirk. It is not simply that a broad concept *has* many paradigms—a broad concept is formed *by* the interaction of these different paradigms. A relatively nuanced, thoughtful concept of fairness will, as we have seen, likely be shaped by both equality of opportunity and equality of outcome. Furthermore, how one understands the relative importance of these types of equality will be shaped by the various contexts in which they are applied, from housing to education, from workplace equality to equality before the law. It will also be shaped by one’s broader understanding of the concept of equality, which is itself shaped by different paradigms, such as what one considers to be the most important categories of equality, e.g. gender, ethnicity, religion. On this understanding of concepts, basic or not, all our concept-words are interconnected in an intricate web of concepts and experiences. That, I think, is why Wittgenstein claims: “When we first begin to *believe* anything, what we believe is not a single proposition, it is a whole system of propositions. (Light dawns gradually over the whole.)” (1977, § 141). But the particular difficulty facing us is that the way in which a specific concept—especially a broad, basic concept—orders and divides these elements into that whole interconnected system is indeterminate, and not given a priori. And yet we do find ourselves thinking according to these concepts.

### 3.3 Intra-conceptual vs inter-conceptual disagreements

These conceptual features lead us to consider a further question that is crucial for clarifying the nature of deep disagreements: how can we distinguish between genuine deep disagreements and ordinary disagreements that happen to be about vaccines or affirmative action. After all, it is surely possible that two people with broadly similar concepts of evidence might still clash over how to interpret certain putative cases of evidence in relation to vaccine side effects. Likewise, as was already suggested in the second vignette in Sect. 3.1, not every disagreement about affirmative action is deep. It seems inherent to moral matters that disagreement over hard cases occurs even where disputants have much in common, conceptually. Given the breadth and the indeterminacy of basic concepts, why should a particular difficult case be treated with special importance?

It is important to look at each individual case to identify the conceptual features on which the dispute turns, in order to distinguish between intra-conceptual disagreement and a genuine deep disagreement, which as I am suggesting takes place between two radically different concepts, e.g. of evidence or of fairness. Ranalli (2021) has suggested that systematicity should be considered a key feature of deep disagreements, and in my view this feature is vital for making this distinction. The systematicity of the difference between rival concepts will determine whether the disagreement is intra- or inter-conceptual.

Consider the case where two scientists have a genuine dispute about the relative importance of case-based evidence in scientific evidence, and whether there is an overreliance on statistical reasoning and randomised controlled trials in contemporary medical practice at the expense of clinical judgement in relation to individual cases—a criticism one finds in the literature on evidence-based medicine (cf. Worrall, 2007; Edwards et al., 2004). The instances of vaccines and autism may well be a test case of evaluating case-based evidence in such a dispute. Here there is sufficient common ground for ordinary argument, and it is likely the dispute about vaccines would not be an either-or case of statistics only or case-based evidence only, but a question of weighting their relative importance.

By contrast, the vaccine-sceptic—if her conceptual difference is truly systematic—is not engaged in a dispute *within* the scientific conception of evidence about the importance of case-based evidence. A rival conception of evidence has been constructed in which statistical evidence is judged as irrevocably flawed and irrelevant in the case of vaccines. This exclusionary character of a rival paradigm is a tell-tale sign that we are in deep disagreement territory; it differs from the disagreement between scientists who have different conceptions of how to accommodate and prioritise different forms of evidence, but do not exclude them completely. In this case, despite some expected overlap between the sceptic’s concept of evidence and the scientist’s, their radically divergent paradigms are entrenched in a systematic way, such that the overlap is ultimately rationally powerless to create the conditions for ordinary argument. Hence it would be unlikely that vaccines are the only context where the scientist and the vaccine-sceptic will have a dispute. As mentioned above, a vaccine-sceptic will adduce in further support of her paradigm of evidence things like the dishonesty of scientists and the possibility of unknown biological factors, and will perhaps look for other cases where statistical evidence has been doubtful. This overall manner of understanding evidence would strike a good scientist as lacking in rigour, prone to cherry-picking doubtful cases and ascribing implausible explanations to cover up gaps. So we would expect to see, as Ranalli (2021, p. 984) puts it, a “*ripple-effect*” where the deep disagreement over vaccines spills over into other related contexts.

It will be similar with the policymaker and the politician in the affirmative action example. They may, in practice, be able to converge on many cases where less is at stake and so work together quite well— this may mask a deep disagreement, or it may be a sign that their differences are less radical than may appear. When it comes to university and boardroom quotas, and other more high-stakes contexts, the depth of their disagreement will be determined by how they characterise their conceptual differences. If the dispute hinges on, say, whether to treat university places as an exception to the rule of not having quotas, then this is not likely to be a systematic difference—perhaps the politician just believes that education, being a social leveller, ought to be the one place where we prioritise equality of outcome. Such a view would still prize equality of opportunity as the ideal, but makes allowances for structural difficulties. But suppose the dispute were characterised in a more *exclusionary* way, where one party views that what is at stake is the very possibility of ever achieving equality of outcome, and that in certain key contexts equality of opportunity is an oppressive tool used to maintain privilege. This would be a sign of a disagreement turning on disputants systematically entrenching different paradigms of fairness.

With these considerations in mind, I will now explore in greater detail the relationship between concepts and reasoning, which will help to further account for the particular depth of deep disagreements.

## 4 Wittgenstein and the nature of reasoning

Having discussed at some length the role basic concepts play in deep disagreements, I will now develop what I take to be an authentically Wittgensteinian account of reasoning and its relation to the formation of linguistic concepts more generally, of which basic concepts of reasoning are a special case. This will give more insight into the particular logical character of deep disagreements—why they are deep and not like ordinary disagreements. As I mentioned in the Introduction, this account is not a reconstruction or an exegesis of Wittgenstein’s view; Wittgenstein’s thought on reasoning is, in large part, scattered over remarks from different contexts and not synthesised into a holistic account. Rather than go into questions of interpretation, I will build up an account that takes inspiration from Wittgenstein’s writings, but does not answer to them.

The central idea I want to put forward in this account is that there is no stable, pure essence of reasoning, which is then shown in its application to different concepts and their contents. Rather, reasoning is inseparable from its mediation by concepts. I find this explanation by Müller to be one of the best articulations of this broadly Wittgensteinian view:

“…these categories [thoughts, considerations, wishes, intentions, decisions, etc.] are—like any other concepts, only, perhaps more obviously—instruments by which we bring division and order into the diffuseness of reality. Without such division and order we cannot reflect on and find orientation in reality. Concepts like *thinking* and *wanting* serve our communication about an otherwise indistinct complex of observable human *behaviour*. Hence it is up to us to define our categories as seems helpful.” (Müller, 1979, p. 107)

This short explanation from Müller contains two points of instruction about how concepts and their formation are constitutive of what it means to reason. The first is that it is characteristic of our concepts that they range over different aspects of some relevant domain in life, but how they range over and order aspects of that domain will depend in large part on the needs and interests of the linguistic community. The second point suggested by Müller is a more difficult one about reasoning and concepts being in something like a symbiotic relationship—we need the “division and order” of concepts to think, but such “division and order”, I suggest, comes from thinking. Let me treat each of these two points in turn.

On the first point, concepts are for Wittgenstein formed not by an intellectual, discursive process but by practical needs and our ways of acting in response to those needs—as Wittgenstein (2009a, § 241) stresses, by “agreement not in opinions, but rather in form of life”. Hence, how we come to form concepts in this way is important not simply for historical reasons, but also for normative ones. As a corollary to this point, it is important to stress that on this view, concepts do not primarily denote a specific entity, whether in thought, physical reality, or some abstract realm—even if, at least in the case of physical nouns, that is how we tend to think of them. Wittgenstein, in the *Philosophical Investigations*, helpfully speaks of words as tools (2009a, § 11). To be more precise for the needs of our discussion, we can think of linguistic concepts as *structuring tools for thought and reasoning*.

Furthermore, the way that concepts serve our interests and needs will not, typically, involve a one-to-one correspondence. “Our interests and needs” is not a discrete set of things that remains constant and is easily individuated. They change, they often grow more complex, and we retain old forms of speaking even as we develop new concepts. So it is common for us to use concepts with overlapping aspects but sometimes with distinct emphases. Nonetheless, our interests and needs are usually made apparent in concept-formation through central or paradigmatic instances of how a concept is to be used in reasoning.

To illustrate using a more neutral domain (neutral in relation to deep disagreements), when talking about musical compositions, we might refer to a “song” or a “piece”—these two concepts will frequently converge, but they represent different, if compatible, conceptual frameworks. On a broader level, between languages and cultures there may be no exact equivalence for the concept “music”. In contemporary British English the word “music” has quite a broad application; certain African languages and North American indigenous languages have no general term for “music”, although the Blackfoot language of North America has something like a general word for music: “saapup”, which means “singing, dancing and ceremony” (Hamilton, 2022, p. 107). We cannot say that Blackfoot speakers have exactly the same concept of “music” as that of British English speakers, but in both languages there exist terms that are used to speak about different aspects of broadly the same domain. Concepts are, nonetheless, not simply about denoting particular items within a set. How each linguistic community’s concept is individuated will depend in particular on its paradigmatic practices of music and related activities (such as cultural or religious ceremonies), and this can also change over time (cf. ibid., pp. 107–111). For example, in a different time period, atonal music might not have been considered “music” in many European languages; historically, poetry has been sung in many cultures, but as poetry and song grow more separate, poetry may now fall into a liminal zone between what is definitely music and what is definitely excluded by that concept. But this is not a question of mere semantics; it invites reflection on deeper questions, such as: what importance does the concept of music have for this linguistic community? Why is it practically helpful to make distinctions between music and non-music (however that distinction is drawn)? These are the normative considerations underlying such concept-formation.

Let us now attempt to tackle the more difficult point that, without the division and order of concepts, we just cannot conduct rational reflection, and yet paradoxically this division and order comes from our need to think. The key to understanding this is the importance Wittgenstein accords to acting; thinking is not a purely mental affair. Queloz (2016, p. 111) observes that in Wittgenstein’s view of concepts and reasons, “what counts as a reason for what is… *causally* determined by—though it need not *logically* reduce to—what we *treat* as a reason for what. It thus rests on our ways of acting…”. This recalls Wittgenstein’s assertion in *On Certainty* (1977, § 204) that “it is our *acting*, which lies at the bottom of the language-game”. In simple terms, reasoning depends on language which in turn depends on how we act. Certain paradigmatic ways of acting carry an implicit ordering of conceptual priorities and boundaries. For example, in a culture which engages in song and dance only for ceremonial purposes and not for entertainment or concerts, such a way of acting implicitly sets normative rules and limits on whatever linguistic concept is formed to articulate thoughts in this domain. At the same time, these paradigmatic uses do not form a ready-made concept of ‘music’ (or functional equivalent) a priori; nor do they provide mechanical criteria or rules for the precise use of these concepts in every possible scenario.^1^ How various paradigmatic instances connect to form a concept is *suggested* but not absolutely determined by our ways of acting. Only in articulating the concept and using it do we actually lay down normative boundaries for its use.^2^

In this way, concept-formation and thinking exist in a bidirectional relationship—one can only think through concepts (already implied by patterns of action), but concepts can only come about when we use them in thought (and this actual use gives them normative significance). As already suggested in Sect. 2, this account of reasoning is more radical than the thought that certain arational commitments *enable* rational evaluation, if that is taken to presuppose some distinction between rule and application. I take Wittgenstein in *On Certainty* to be rejecting this conventional distinction when he says that there is no sharp boundary between them (1977, § 319). Concepts are not formed in order for reasoning to take place, which would be a simplistic picture of rule and application. Concept-formation and reasoning are symbiotic. Without a relevant concept, we could not even reason about a particular domain of life; how we act in that domain does not force a concept on us, but suggests one; but for a concept to be meaningful it must have normative rules for use (i.e. there must be correct and wrong uses, otherwise it would be meaningless); but in the use of concepts, its rules are continually formed, solidified, and sometimes even modified. So to even possess a concept is already to have substantial structures of reasoning in place, and not simply fixed points for further inferences.

Another passage from *On Certainty* is significant in helping us understand the symbiosis of reasoning and concepts:

“All testing, all confirmation and disconfirmation of a hypothesis takes place already within a system. And this system is not a more or less arbitrary and doubtful point of departure for all our arguments: no, it belongs to the essence of what we call an argument. The system is not so much the point of departure, as the element in which arguments have their life.” (Wittgenstein, 1977, § 105)

Our background system of concepts is not the “point of departure” of rational agreement and disagreement; our system of concepts, as we have seen, is already alive with reasoning on the basis of our ways of acting. In this way, concepts are normative structures of thought and reasoning.

Let us illustrate this normative structuring by returning to the relatively neutral territory of music. Consider two fictional languages and their main term for music, which have slightly different extensions:

Music_A_: In Language A, the term for music encompasses all forms of singing—folksongs, art songs, chanting, and even poetry recited rhythmically—but excludes purely instrumental compositions.

Music_B_: In Language B, the term for music encompasses all forms of tonal composition—vocal, instrumental, or both—but excludes purely rhythmic compositions, with or without words.

We would recognise both Music_A_ and Music_B_ as concept-words for “music” as we, e.g. speakers of British English, understand the term. It would be tempting to think that both languages have an incomplete conception of music—why do they not have a general term for the wider domain of music, comprising all forms of auditory compositions, whether tonal, atonal, rhythmic, or some combination of them? But from the perspective of a speaker of either fictional language, what we consider “the wider domain of music” just does not exist in their thought and reasoning. Perhaps in the linguistic communities of both Language A and Language B, there has been no practical need (e.g. artists wanting to form unions to fight for better rights) to use a term that ranged over all auditory compositions. Whereas for convenience of explanation I previously spoke of how concepts range over aspects of their relevant domain, it is important to see how the “relevant domain” in question is not pre-existing, waiting to be divided up and ordered into our linguistic concepts—the relevant domain exists only as a result of, and in, the division and order of concepts. To speak of radically differing concepts like Music_A_ and Music_B_ as occupying “the same” domain is a helpful way of speaking in cases where there is sufficient conceptual overlap (which does not preclude systematic differences), but it is not an unqualified statement because there is no domain given prior to either concept.

This fictional example thus helps us see how it is only through the use of these concepts, in response to needs and interests, in their respective linguistic communities that reasoning is possible and acquires a normative structure. We return to the point regarding the blurred distinction between rule and application, leading to a symbiotic relationship between reasoning and concepts. This is also where hinge commitments come into the picture, although I have a different understanding of their role from Pritchard’s. Notice that speakers of Language A and Language B will have many overlapping hinge commitments about music. If they were listening to a CD of classic English hymns sung by King’s College Choir, it would be a hinge commitment to say “this is Music_A_/Music_B_”, just as it would be for us to say “this is music”. Relative to their particular concepts of music, none of these hymns would require *proving* by means of evidence or further evaluation that they were instances of Music_A_/Music_B_. They are paradigm cases of music in both languages. Doubt in this case would be unintelligible (cf. Wittgenstein, 1977, §§ 2;4;10). However, speakers of Language A and Language B would have a deeply entrenched dispute about whether poetry or rap counted as Music_A_/Music_B_ or not. This would be a systematic difference, as shown in the way they reason about other related matters. For example, speakers of both languages would likely have a different understanding of the relationship between music and literature. Perhaps in Language A, Music_A_ is a subset of Literature_A_, while in Language B the distinction between Music_B_ and Literature_B_ would be more similar to the distinction between music and literature in British English. Or suppose speakers of Language A saw someone through a window fiddling with what looked like a foreign musical instrument. “Do you think he is making Music_A_?” one of them asks. “No, I don’t think so, because I can see he isn’t singing.” Someone else with the concept of Music_B_ would, no doubt, reason about the matter completely differently.

In summary, we can say three things about the relationship between concepts, concept-formation and reasoning. Firstly, the division and order imposed onto reality by our conceptual categories is not neutral. Our concepts create a normative structure for thought and reasoning, and they do so in a context of indeterminacy. We—as in British English speakers—might think that it is natural for the concept of music to range over all forms of auditory compositions, but that is only because we are presupposing *our* concept of music. In another language or culture, this idea may not be intuitive because concepts have divided up reality differently. We do not know the histories of the two fictional languages and their respective linguistic communities, but we can surmise that each community will have its particular cultural and social practices which have shaped their respective concepts of music.

Secondly, that there is substantial overlap between concepts is not surprising, because concepts are suggested by our ways of acting though they do not force themselves on us. Without certain hinge commitments—paradigmatic instances we readily identify as falling under the concept—we would not even have that concept in the first place (cf. Wittgenstein, 1976). In this way, the line between rule and application is blurred. The concept of music depends on the natural fact that there are some combinations of sounds which strike us as being meaningfully different from mere noise. Certain domains, or aspects of a domain, in life seem to naturally cluster together in linguistic concepts across different languages, though we cannot take this for granted a priori—it would be odd, but certainly not impossible, to have a concept that only included music and visual artforms, but not literary art.

Thirdly, although I have only used the extension of Music_A_ and Music_B_ to illustrate their differing normative structures, these have already helped illustrate the extent to which concepts structure reasoning before we even begin to make further inferences on their basis. In real, more complicated cases—such as in the deep disagreements we have been considering—it is more than the range of things picked out by a concept that accounts for its normative structuring, but also the way it orders and prioritises elements, sometimes to the exclusion of others, and evaluates their explanatory power. A shared domain (which is not given a priori) between radically different concepts may allow for some dialogue, but is no guarantee of the possibility of ordinary argument.

For these reasons, the relationship between concepts, concept-formation and reasoning is a complex, symbiotic one. What is clear is that in pointing to constitutive concepts of reasoning as the locus of deep disagreements, we are not pointing to a foundational layer for the rest of reasoning, but to rather substantial normative structures of reasoning. This is why “depth” or “foundation” metaphors can be misleading, and as discussed in Sect. 2 they can lead to explanations being insufficiently fine-grained. Wittgenstein’s well-known hinge metaphor in *On Certainty* (1977, §§ 341;343) is a kind of “depth” metaphor—the door (rational evaluation) cannot turn without the hinge (our optimally certain convictions, which cannot be doubted), which now appears to be a deeper, foundational layer. But he gives another, more arresting metaphor which collapses the rule-application distinction that “depth” metaphors might suggest:

“I have arrived at the rock bottom of my convictions.

And one might almost say that these foundation-walls are carried by the whole house.” (ibid., §248)

So even when we talk of basic concepts of reasoning like evidence and fairness, to which we will return shortly, these concepts are “carried by the whole house” of the practices and applications built on them.

## 5 Deep disagreements and the radical indeterminacy of concepts

The account I have supplied in the last section should make clear, by now, why deep disagreements have a particular “depth”.

First of all, given that they engage concepts that are constitutively basic to reasoning, and given what we have now seen about the importance of concepts to any kind of reasoning, we can more definitively say that deep disagreements are disagreements that involve clashes between competing normative structures of thought and reason. These competing normative structures cannot appeal to further concepts since they are indispensable to broad swathes of reasoning.

This perspective also offers a more fine-grained manner of understanding deep disagreements. To point to the role of basic concepts is not to point to one fundamental, separable layer in our reasoning or belief system, given the breadth and the indeterminacy of our concepts, and the way they are bound up with the interests and needs of their communities. So we must understand that different people develop radically different basic concepts, whether of evidence or of fairness, often not solely in response to intellectual considerations but in response to their life experiences. Somebody who has witnessed what they perceive to be side effects of a vaccine on a loved one, or somebody who has personally experienced disadvantaged circumstances resulting from historically entrenched systems of oppression, will likely form concepts on the basis of these different paradigms. Recognising the symbiosis of reasoning and concepts should prompt a certain intellectual humility among all of us, for however well thought out we think our positions are we cannot (and arguably should not) totally escape our personal and communal histories.

Furthermore, the dangers of the “depth” metaphor, as discussed just a moment ago, sheds new light on the systematicity of deep disagreements. Because Wittgenstein has collapsed the distinction between rule and application, we cannot understand deep disagreements as a matter of contested applications of a concept. A borderline case of affirmative action, like special training programmes or financial support for disadvantaged groups that stops well short of quotas (cf. Taylor, 2009) may be an instance of interpretation and application of the policymaker’s concept of fairness, in order to accommodate such a concession within her conceptual scheme. Likewise, there may be difficult cases in medical research where the relative weighting of statistical evidence and case-based evidence is unclear. But deep disagreements will typically involve what is a paradigm case of the contested basic concept for one or both parties in the dispute. So the disagreement is transposed to the level of concept-formation. Disputants in an affirmative action deep disagreement do not ask, “is this right within my concept of fairness”, but rather, “is this my concept at all”? Likewise with evaluating vaccine safety, the scientist and the sceptic are both clear about what counts as valid, indeed paradigmatic evidence according to their respective concepts of evidence.

But despite the depth of deep disagreements now being established in my account, we have to tackle another question: are they actually disagreements? This is not a new question in discussions of deep disagreements (see, for example, Lavorerio, 2021a, pp. 221−224; Godden and Brenner, 2010, pp. 46− 47). And as I mentioned in Sect. 2, this question is of profound importance for understanding deep disagreements, for there is a potential dilemma: either deep disagreements are completely irresolvable by rational means, and so are probably not actual disagreements, or they are resolvable, in which case the category of deep disagreements is called into question. Perhaps they are just difficult disagreements, but not logically or epistemologically special. Through my account of concepts and reasoning, I have sought to show that there is something logically special about deep disagreements. I will now demonstrate how deep disagreements are still genuine disagreements, thus resolving this dilemma.

### 5.1 Deep disagreements as disputes in reason

There is a good Wittgensteinian case to be made that deep disagreements are not genuine disagreements— at least, this seems to be the case on the surface. The question of whether the opposite of a proposition is intelligible was always a significant one for Wittgenstein, from the *Tractatus* right through to *On Certainty*. Doubt is only meaningful where we have some conception of what it would be like for the proposition being doubted to turn out to be false (Wittgenstein, 1961, 6.51; Wittgenstein, 1977, §32). By this measure, we should not call an argument a disagreement unless agreement is in principle possible. It would also seem like a disagreement is only genuine if the disagreement concerns reasons. We would not call a mere conflict of emotions about a situation, for example, a disagreement; we expect genuine disagreement and resolution in agreement to be a rational matter. Finally, in a genuine disagreement the disputants should be concerned about substantially the same matter, both in the subject of the dispute and in their approach, at some level. Sometimes disagreements, deep or shallow, appear like people merely talking past each other—is that truly disagreement? I would think not.

Fogelin’s own account seems to suggest that deep disagreements are just that—people talking past each other—since he talks about “the conditions for argument” being non-existent (1985, p. 5). On Pritchard’s account, as we have seen earlier, deep disagreements involve divergent hinge commitments, but since these are the arational grounds of the disputants’ respective rational evaluations, this is not a disagreement in any sense relating to reasons. At most, this may be an instance of faultless disagreement, since both disputants are not wrong relative to their local rational evaluation. Ranalli (2020, pp. 4985−4986) raises this criticism against another hinge epistemology approach to deep disagreements, but I think it applies equally to Pritchard’s. On the other hand, Siegel (2019, p. 1113) rejects hinge epistemology in part because he sees it as rendering genuine disagreement impossible in putative cases of deep disagreement, and yet he thinks that we clearly do disagree about these matters. Which view is right?

On the Wittgensteinian account of reasoning I have developed, a deep disagreement would be rational disagreement because the disagreement is caused by a radical difference in concepts, and not just hinge commitments. I agree with Pritchard that hinge commitments—which I have accommodated into my account—are indeed arational and non-epistemic commitments. But concepts like music, evidence, fairness, and God are tools for rational evaluation. They are, as Wittgenstein says, elements in which arguments have life—they do not *enable* rational evaluation, but rather rational evaluation is nothing if not the use of such concepts. So a dispute concerning concepts is still a dispute in reasoning, so long as the dispute is still substantially of the same matter.

But do deep disagreements really concern substantially the same matter? Is there enough common ground to call it a genuine disagreement? On the basis of my account of reasoning in Sect. 4, I can answer in the affirmative. Essentially, despite the difference in conceptual paradigms between disputants in a deep disagreement, the fact that the disagreement can still be understood as being a dispute about how to form concepts within the same domain allows it to be a genuine disagreement. That there can be overlap in other contexts between radically different conceptual paradigms, as we saw in Sect. 3.2, and that concepts clash because they are different normative structures about substantially the same domain, speak to this possibility of genuine disagreement. The clash of deep disagreements involves irreconcilable attempts to bring order and division to the same or at least overlapping elements of reality. And that is why there is a paradoxical air to deep disagreements: one party can see the other’s concept as a rival one of evidence or fairness, and simultaneously thinks that the rival concept is so far off the mark it cannot really be considered evidence or fairness.

Recall that the fictional examples of different music concept-words were artificially simple to bring out the conceptual point, such that the difference between concepts was primarily a difference in extension, i.e. what was included and what was excluded. In many deep disagreements, the difference in concept may not simply be in extension, but also in the prioritising of elements and an evaluation of their explanatory importance. Consider, for example, a common criticism of Dawkins’s New Atheism: Dawkins argues that God must be a complex being, and it is this notion of God he strives to disprove. But classical theism posits that God is a being of perfect simplicity, so Dawkins is not disproving the existence of the God of classical theism (see, for example, Taylor, 2013 and Plantinga, 2007). If this was all there was to disputes between classical theists and New Atheists, then this would not be a genuine disagreement. The same concept-word happens to be used about two rather different purported beings. But the relevant domain of the concept of “God”—for both believers and atheists—tends to be much wider than that. An atheist may still object to the idea of the universe having a creator, or may object to the existence of God on the basis of the existence of evil. The difference between having a concept of a necessarily existent God and having a concept of an impossibly existent God is indeed a systematic one, involving substantially the same aspects of the domain(s) of the divine, the spiritual, and the transcendent, but differing in their evaluations of explanatory power. This is a more acute form of normative structuring than mere extension.

This point applies equally to the disputes over the concepts of evidence and fairness we saw in my two examples of deep disagreements. Here we can say it is the latter pair of features from Sect. 3.2—breadth and indeterminacy—that are crucial to understand how deep disagreements are still about substantially the same thing. But this point also leads us to consider how concepts of practical reasoning suffer from a far greater problem of indeterminacy, and I wish to explore this issue for a moment.

### 5.2 Radical indeterminacy in moral concepts

If we consider the case of vaccine-scepticism, we can see that although the scientist and the vaccine-sceptic have such different concepts of evidence that one cannot be corrected from the other’s internal standards, these different concepts are normative structures imposed onto substantially the same domain. It is therefore significant that the vaccine-sceptic’s reported incidents of close connections between jabs and autism symptoms developing are not, in fact, irrelevant to a proper scientific conception of evidence. This is a sign of the breadth of the concept’s domain. Absent other evidence or explanations, a good scientist ought to take reports of such incidents seriously. They may well be indications of an as yet undiscovered causal relation. But what a good scientist does is also to weigh evidence according to the rigour of the methods by which they were collected. It is possible that all the other available evidence, in the form of statistical data about vaccines, the best understanding of potential biological mechanisms or pathways for jabs to cause autism, and our accumulated knowledge about when symptoms of autism are first spotted, may fail to capture this putative causal relation, but it is so unlikely that those initially compelling incidents cannot count as scientific evidence. The scientist’s situation is like that of Wittgenstein’s example in *On Certainty* of whether it is possible to doubt that I have a brain. “Everything speaks in its favour, nothing against it”, despite it being *imaginable* that I do not have a brain (Wittgenstein, 1977, § 4). The scientist’s concept of evidence does not rule out imaginative possibilities of doubt, but it does not take them seriously either.

By contrast, for the vaccine-sceptic the faint plausibility of her paradigmatic incidents of evidence allows for a radically different concept to be developed. Since the purported causal relation in such incidents cannot be totally disproved, they lead to a certain indeterminacy in the concept of evidence. Such imaginative possibilities take centre-stage as *the* paradigm of a different concept in the same domain. This, I think, is also what happens with flat-earthers, who give a completely different priority to what a scientist would consider as having little explanatory power or having the remotest possibility of being true. The rival, non-scientific conception of evidence at work takes the immediately observable connection to be true until disproved, which is a radically different prioritising of the elements of the concept of evidence from the criterion of falsifiability—the notion that a hypothesis is a valid scientific one because it can, in principle, be disproved.

Yet I think most of us will quite quickly recognise that the vaccine-sceptic’s or the flat-earther’s concept of evidence is—if we cannot call it the “wrong” concept—a much weaker concept in its explanatory power. But in moral matters, even where we have a strong conviction, say, of the wrongness of affirmative action, I suspect we will not usually be so quick to dismiss our interlocutor’s concept as obviously “weaker”, if we are trying to be good moral philosophers. Why is that so?

The existence of imaginative possibilities of doubt may be more easily dismissed in concept-formation pertaining to physical matters like “evidence” and “causation”, though it speaks to the indeterminacy of concept-formation. But, I would argue, concept-formation in practical reasoning does not fall back on a substrate of *physical immediacy*. A scientist’s conception of evidence and causation is a complex one, but as Anscombe argues in her lecture “Causation and Determination” (1981), the concept of causation still has as its root the ordinary, physically immediate forms of causation like scraping or digging which, to adopt Wittgenstein’s phrase, would be “exempt from doubt” (1977, §341). With moral concepts like fairness, there is no direct equivalent.

That is not to say that we would not have hinge commitments of fairness. Following the account of reasoning in Sect. 4, we would say that there is no arguing about fairness at all until we have some concept, some elementary distinction at least, of fairness and unfairness. Fairness, as I have suggested, is the concept by which we make sense of what we owe to others—which is an incredibly broad domain. Our policymaker and our politician, who have such divergent conceptions of fairness, may well share many hinge commitments of fairness. Surely, no one who looks at past histories of oppression will say “this is fair”. Conversely, the politician—despite being an advocate of affirmative action—may not be so radical as to think that the purchase of housing must be subject to strict quotas. Perhaps this is a paradigmatic instance of where the politician and the policymaker agree, and both parties will thus share hinge commitments about what is fair in relation to providing prospective homebuyers equality of opportunity. This is another example of how concepts are “carried by the whole house” of different elements and applications. But how we prioritise the importance of these different aspects of the domain of fairness will affect the normative structuring of our concept of fairness, and just one or two particular contexts of applying these concepts may expose their systematic differences. In concepts pertaining to the physical world which we use in theoretical reasoning, these concepts can be refined with methods of empirical testing. So we might have an elementary understanding of “water” even without knowledge of its molecular composition, but scientific discoveries and methods of testing give us more sophisticated paradigms of the concept. By contrast, with concepts of practical reasoning there is no such empirical testing of their validity. The indeterminacy of their normative structuring is therefore different in kind.

We have, therefore, quite a substantial account by now about how deep disagreements are disputes in reasoning, and I have shown that deep disagreements are disputes about substantially the same matter.^3^ This argument has also revealed a particular difficulty faced by concepts of practical reasoning, which may be the source of the feeling that moral deep disagreements are particularly intractable. But I have yet to discuss whether deep disagreements are in principle open to the possibility of agreement—and, importantly, agreement that is recognisably rational, and not due to force.

### 5.3 Pragmatic sources of rational agreement

The agreement that is possible in the face of deep disagreements must, I think, be explained in terms of the use of our concepts. The account of concepts and reasoning I have given is one that is somewhat pragmatic in nature—concepts serve our purposes, and their formation is therefore bound up with our interests and practices. As I suggested, the community of speakers of Language A may not have had reason to develop a term that ranges across all forms of auditory compositions. This line of thinking is suggested by some remarks late in the *Philosophical Investigations* (2009a, §§ 466–70), where Wittgenstein asks why humans think—do we think, reason and calculate because it pays us to do so? How do our concepts serve us? If this is the right way to think about concepts, then I think agreement in concepts is also to be evaluated in terms of how it serves our interests.

Let me now take the fictional example of music concepts as a counterexample. Recall that between Music_A_ and Music_B_ there will be a dispute about whether poetry and rap count as music. Is this a meaningful dispute? So far, nothing in my account would suggest otherwise. However, I would suggest that this dispute is meaningless—artificial, at best—if nothing hangs on this potential agreement. It would be like a casual argument between friends about whether it is intrinsic to the concept of “cake” that it is baked or whether ice-cream cakes are excluded. Is there a reason why it is important to settle the status of poetry and rap? Perhaps it matters because it relates to eligibility conditions for a prestigious artist’s prize in a community where both Language A and Language B are widely spoken. This might turn the dispute into a somewhat meaningful disagreement, although the relative importance of this dispute in the grand scheme of things is possibly still rather low. It is also dependent on a very contingent set of circumstances.

Disputes about vaccines and affirmative action, by contrast, are generally regarded as hugely important. They touch on endeavours and areas of reasoning that can make a huge impact on people’s lives. Of course, the contexts of dispute are always going to be contingent, but because the concepts of evidence and fairness are so important to human life, the importance of achieving a higher degree of convergence in concepts transcends the specific contexts of disputes. This is unsurprising given their indispensability and unjustifiability. Having a conceptual category of music, by contrast, does not seem essential to reasoning well (this does not imply that the *practice* of music is inessential to human cultures).

So the question of what agreement would look like in deep disagreements has to be tackled by asking why people might change their minds about vaccines or affirmative action—what would happen that would make them realise they needed a new concept? When it comes to this level of concept-formation, this is as rational as it gets. One can imagine that our moderately Rawlsian policymaker may change her mind about affirmative action after a prolonged study of historic oppression in her country and engagement with victims of that oppression. That is the kind of activity that often leads to a change in moral paradigm. But conversely, the politician may also undergo a change of heart after being repeatedly questioned by those who become the new victims of affirmative action policies. Broadly speaking, we have to be confronted by a situation or series of situations that makes us realise *the inadequacy of our normative structuring* of the world, with respect to needs and interests we have hitherto neglected or not known.^4^ This is what I would consider extraordinary means of rational resolution—the ordinary marshalling of reasons will not usually achieve this, but over time they may create the *conditions conducive for such a realisation* of inadequacy.

This is important because we cannot change our concepts at will. Although concepts are what they are because of their usefulness to us, this does not mean we can simply go concept-shopping and choose to adopt new concept just like that, on account of its usefulness. This would, first of all, be dishonest and opportunistic.^5^ But there is another reason why this would also be *irrational*: concepts are not for personal use only, but they are shaped by the different practices and experiences of the linguistic community as a whole, into which we are inducted. So there is a community-wide context to our background concepts which suggests a further thought, that reasoning is intrinsically an activity of a community. The “community” in this sense does not mean all speakers of my language, but a virtual community of those who share the same paradigms as I do, shaped as I have been by relevantly similar experiences. We reason always as part of some community, with our needs and interests made intelligible to us through that community’s linguistic standards. That is why although, from a view-from-nowhere standpoint, it will look as if our concepts are arbitrary, because we are individual reasoners are tied to our linguistic community we cannot change our concepts arbitrary—at will, as the German *willkürlich*, makes clear the meaning of the word. That is why I think Wittgenstein stresses that, in this sense, our concepts are not arbitrary:

“Compare a concept with a style of painting. For is even our style of painting arbitrary? Can we choose one at pleasure? (The Egyptian, for instance.) Or is it just a matter of pretty and ugly?” (Wittgenstein, 2009b, § 367)

And that is why any change to our concepts will likely be gradual, organic, and holistic. It is not so much changing a concept, as undergoing a change in the community or communities with which I make sense of my life, and come to see the inadequacy of my previous way of reasoning. There are no a priori criteria for perceiving the inadequacy of our concepts because of the intersection between concepts and experience as I have been stressing so much. But it does not mean that this perception of inadequacy is not a rational one. It is difficult to capture because it concerns the conditions for the formation of concepts, which as we have seen are bound up in an interconnected web of further concepts, practices and experience. But if agreement *is* achieved in deep disagreements, it will be agreement in a significant area of reasoning well, in a way that agreement about the conceptual category of music is likely not so significant to reasoning as such. That is what agreement in a deep disagreement, in principle, looks like.

I believe there is a final, brief reason why this manner of resolving deep disagreements should be considered rational: it is not all that different from how ordinary disagreements are actually resolved. I do not mean disagreements where one party is factually wrong, where all that is required is verification through mutually acceptable reliable sources. Two politicians in favour of a programme of affirmative action may, for instance, disagree on the precise extent or speed of the roll-out of their policies. These differences need not be anything deep and systematic, but may be the result of the age-old tension between pragmatism and idealism. How are such ordinary, but potentially persistent, disagreements resolved? The marshalling of reasons does not mechanically shift one’s opinion. Perhaps the more pragmatic politician suggests that a slow roll-out, though limiting effectiveness, will in the long-run better win over the electorate to such a radical programme. The idealistic politician may take a while to come round to this viewpoint, but perhaps after more consultation with voters will reassess the importance of winning over hearts and minds relative to fighting injustice as soon as is feasible. What is involved in what we often call a “change of heart” is difficult to characterise in rational terms—it is a subtle shift in one’s conceptual ordering between pragmatism and idealism, not unlike the more dramatic shifts required to resolve deep disagreements.

In summary, we can say that deep disagreements genuinely are disagreements, and their “depth” does not render them in principle irresolvable.

## 6 Defusing relativism with the limits of reason

In this paper, I have put forward an account of reasoning and concept-formation that explains the nature of deep disagreements, with special regard for the particular difficulties of concept-formation in moral matters. Deep disagreements, on my account, are essentially disputes about what it means to reason well, involving radically different paradigms of a constitutively basic concept of reasoning. The pragmatic nature of concept-formation, on my account, explains on the one hand how deep disagreements could be resolved, but on the other hand also accounts for the serious difficulties involved.

For reasons of clarity, I have used examples that highlight a clash in one particular basic concept per disagreement, but there are certainly deep disagreements that engage more than one basic concept. Abortion and euthanasia are obvious examples—they seem to involve clashes concerning the concepts of autonomy or freedom, as well as the concepts of justice or care. This is possible because nothing in my account of basic concepts suggests that they are individuated from one another. Very frequently, even basic concepts are formed in part by interaction with each other. Concepts of fairness, freedom, and care are interlinked. Hence, my account of deep disagreements can still be used to explain the nature of such multi-concept deep disagreements. The basic question remains: what are the basic concepts involved, and what are my paradigms for this concept, and how do they radically differ from my interlocutor’s? Movement towards agreement begins when one is able to ask: have my concepts been inadequate in any way, for example in failing to account for a particular type of suffering or a particular duty of care?

Nonetheless, in my account of what agreement in deep disagreements look like, there might be what looks like a disquieting suggestion of a relativism of reasoning, since one cannot really ask what is the “right” concept to have. In concepts used in theoretical reasoning, these at least can often rely on empirical testing as a kind of scaffolding of reliability, on which concepts can be further refined and developed. But there is no such empirical scaffolding in moral concepts, even if there will often be shared hinge commitments or intuitions. Hence, the fear of relativism is particularly acute in the case of practical reasoning.

To conclude the argument of this paper, I want to make some suggestions towards defusing this threat of moral relativism. This discussion, though brief, will also give added perspective to the question of how rational agreement can be achieved in deep disagreement.

The first step is to ask whether my account truly leads to a relativism of reasoning, theoretical or practical. Certainly, in my account I am committed to the view that *human* reasoning is dependent on *human* needs and interests. This looks like a kind of relativism if we think that there must be an extra-human criterion for judging the correctness of human reasoning. But human reasoning is meant to serve our needs and interests, then this dependence is not in itself a cause of relativism. It points to an internal criterion for human reasoning.^6^

This on its own does not settle the question of relativism. We have already seen that human needs and interests are varied, over time and between communities, and that it can take time to realise the inadequacy of one’s own concepts. Does this inadequacy not presuppose some conception of the “right” or at any rate a “better” concept? How could that be made sense of in the context of my account of concept-formation and reasoning?

Once again, it helps to start with a non-moral example. Godden and Brenner suggest, along very similar lines to my account, that normal disagreements are more like “disagreements about measurements (the application of a concept), while “deep disagreements arise from differences in measures (the determination or adoption of concepts)” (2010, p. 49). A deep disagreement would be like a clash between irreconcilable systems of measurement—but, as per my argument in Sect. 5.1, this clash must still be about substantially the same matter, i.e. measurement in this case. Measurement, once again, is a fairly basic concept to reasoning—it seems indispensable to extremely ordinary situations. Comparisons of size or distance are basic linguistic tools of measurement; of course, we have more fine-grained measures for different purposes, and disagreements may break out over what is the best way of measuring a particular phenomenon (e.g. how to measure immunity from a virus), or what it means to measure something at all—the coastline paradox being a prime example here (cf. Mandelbrot, 1967). But even in disagreements about measurement that may well be deep, they are still recognisably disagreements about *measurement*. That is to say, the *concept* of measurement (as opposed to its domain in reality) appears to have some logical limits. We can recognise divergent systems of measurement as measurement, but a system that was too arbitrary or with no fixed points of reference, for example, would just not count as a form of measurement.

I suggest that we can say something similar about concepts of practical reasoning, and so defuse the threat of a moral relativism. It is admittedly more difficult to ascertain which concepts of practical reasoning are indispensable, especially given the wide variety of human needs and interests that our concepts serve. But we can start with what is most obvious—there are needs for which nobody is required to give reasons to fulfil. Food, shelter, clothing, and hygiene come to mind. We do not think of them as instrumental to welfare so much as providing logical limits of some broad concept of welfare or care. Seeking companionship in friends and family—again these are not so much instrumental to a good social life as the very paradigm of it. Speaking and listening are conditions for the concept of communication, which implies truth-telling. There may be some arbitrariness as to how we divide and order these elements of reality, as always, but these concepts speak to real, not arbitrary needs. So the question of what concepts are basic to practical reasoning looks empirical, but it really is a logical question, and this seems in keeping with Wittgenstein’s blurring of the distinction between rule and application in concept-formation.

So in the final analysis, we can understand concepts of practical reasoning in this way. They are normative structures with which we advance our practical needs and interests. These needs and interests may grow more sophisticated as culture develops and society changes, but at root they do not (at least in the foreseeable future) radically alter our most indispensable needs. While our moral concepts are not *justified* by our basic and regular human needs, they are in some way answerable to them. The development of our moral concepts—of autonomy, welfare, honesty, and so on—to encompass more and more complex social realities is always a work in progress, but as long as there are logical limits to these concepts, then there is no global relativism of morality. I suggest that, with respect to those needs for which we typically do not ever need to give reasons for fulfilling, these are indeed limits to practical reasoning. It is in reflecting on these limits—asking whether some rival concepts are indeed forms of “welfare” or “honesty”, for example—that we may even begin to resolve some moral deep disagreements.

## Footnotes

1 This, surely, is one of the principal lessons of Wittgenstein’s rule-following considerations in the *Investigations* (cf. 2009a, §§ 143−242).

2 Although this view of concept-formation resembles what is called the prototype theory of concepts in psychology, Glock (2010, pp. 89−90) points out that prototype theory essentially aims at an empirical and psychological explanation of concepts, whereas Wittgenstein’s account of concepts aims at a normative explanation.

3 See Godden and Brenner (2010, pp. 46−47) and Kusch (2013, pp. 38−39), for other accounts of why deep disagreements still involve much shared ground, which makes them proper disagreements.

4 “To be ‘won over’… involves accepting a certain picture of the world; it involves learning to apply concepts in a way to which one was, perhaps, not initially inclined, and then ‘recognizing’ (understanding, judging, appreciating) that *this* use of concepts is befitting of one’s projects—it ‘allows one to go on.’ The resultant conceptual shift will involve a new understanding of things it will be holistic rather than singular—commonly it will involve broad reaching changes in one’s activities…” (Godden and Brenner, 2010, p. 77)

5 When brain death was first mooted as a new paradigm for defining death in Harvard, this was met with criticism that the change was opportunistic, to enable organ transplantation—although the historical record seems to cast doubt on this being the intention of the Ad Hoc Committee of the Harvard Medical School to Examine the Definition of Brain Death (See Belkin, 2003).

6 See Pears (2006, pp. 17−36) for a discussion of how Wittgenstein’s philosophy gives a new twist to Protagoras’s dictum, “Man is the measure of all things”.
